# Should lifelong anticoagulation for unprovoked venous thromboembolism be revisited?

**DOI:** 10.1186/s12959-015-0063-z

**Published:** 2015-10-05

**Authors:** Otto Moodley, Hadi Goubran

**Affiliations:** Saskatoon Cancer Centre and College of Medicine, University of Saskatchewan, 20, Campus Drive, Saskatoon, SK S7N4H4 Canada

**Keywords:** Venous thromboemobolism, DVT, PE, Recurrence, Duration, Warfarin, NOACs

## Abstract

Venous thromboembolism [VTE] is a common medical condition that has significant morbidity and mortality. Although stringent guidelines recommend lifelong anticoagulation for patients with unprovoked VTE, the optimal management strategy for their long term treatment remains controversial.

Whereas in cancer-associated VTE and second unprovoked VTE lifelong anticoagulation is universally accepted, a careful analysis of the benefit vs. risk of long-term anticoagulation following a first unprovoked VTE should be considered as case fatality rates [CFR] from VTE appear more pronounced in the first few months. The CFR from major bleeding remains constant throughout therapy. Therefore, the risk of bleeding may be underestimated over longer treatment periods relative to the morbidity of recurrent VTE which appears to peak in the first year. The current review highlights the balance between the recurrence risk and bleeding risks in the era of direct oral anticoagulants.

Vitamin K antagonists have been the standard of care for over 50 years bearing significant bleeding risks. The new oral anticoagulants [NOACs] have shown similar efficacy and perhaps a questionable improved safety profile when compared to warfarin.

Aspirin has historically not been a useful agent in the management of VTE. However, two recent trials [WARFASA and ASPIRE] showed a likely 20-30 % risk reduction when compared to placebo for recurrent VTE after initial anticoagulation. The risk of major hemorrhage was low in both trials.

With the emergence of NOACs and the increased utility of aspirin, there are multiple therapeutic options for long term management for VTE. Given comparable efficacy and improved safety of NOACs and aspirin, the risk benefit of anticoagulation is improving.

A risk stratification model may help identifying patients at high risk for recurrence necessitating a lifelong anticoagulation. This cohort should be separated from a low risk group that may benefit from clinical observation, aspirin or NOACs.

Prospective clinical trials are needed to support these clinical observations.

## Introduction

Venous thromboembolism (VTE) occurs for the first time in ≈ 100 persons per 100,000 each year in the United States, and rises exponentially from <5 cases per 100,000 persons at the age of 15 to ≈ 500 cases (0.5 %) per 100,000 persons at 80 years. Approximately one third of patients with symptomatic VTE manifest pulmonary embolism (PE), whereas two thirds manifest deep vein thrombosis (DVT) alone [1].

VTE provoked by transient risk factors is associated with a lower risk of recurrence necessitating 3–6 months of anticoagulation whereas unprovoked VTE has a much higher recurrence risk and its treatment remains challenging necessitating more prolonged secondary prophylaxis [2].

Although, most guidelines advocate for long term anticoagulation for patients with unprovoked VTE [3, 4], advising patients and referring physicians about the optimal duration of anticoagulation after acute unprovoked VTE remains a very common PE/DVT consultation in the outpatient setting. Because extending anticoagulation for an additional three to nine months does not result in further long-term reduction of recurrences following the discontinuation of anticoagulation, indefinite treatment duration should be reconsidered. However, case-fatality rate for major bleeding in patients taking warfarin for more than three months is higher than case-fatality rate of recurrent VTE. Anticoagulant therapy is accordingly a double-edged sword; evaluating patients is therefore complex as it requires balancing the risks of recurrent VTE in the absence of anticoagulation against the risks of bleeding complications with continued pharmacological therapy [5]. The duration of anticoagulation in this context is based on discussion with our patients about their preferences and the strength of the communicated message which is often nuanced [6]. Physician knowledge, attitudes, and beliefs are therefore partially responsible for the gap between actual practice and international guidelines [7].

Worldwide, vitamin K antagonists [VKA] such as warfarin remain the predominant anticoagulant prescribed for VTE [8]. In 2010 alone, for example, more than 25 million prescriptions were filled for warfarin in the USA [9]. Its multiple interactions with food and other medications and its narrow therapeutic range renders it a difficult agent to utilize properly, being responsible for one-third of emergency hospitalizations due to adverse drug events in patients 65 years of age or older [10]. Not surprisingly, in view of its actual and perceived bleeding risks, warfarin continues to be largely underused in clinical practice [11]. A recent study highlighted this underutilization; in a 1-year adherence study conducted on 8040 VTE patients identified as being at high risk of recurrence (mean age 61 years, 59.4 % male), 76.9 % were not compliant with warfarin therapy based on the proportion of days covered (more than 80 % of days) and 51.5 % discontinued therapy [12].

With the advent of the new oral anticoagulants [NOACs] characterized by their rapid onset of action, predictable pharmacokinetics and anticoagulant effect, specific coagulation enzyme target and their low potential for drug or food interactions,[8] the risk benefit ratio may apparently be more leaning towards benefit offering a potential for a safer long-term anticoagulation. NOACs are more accepted by patients (especially the elderly) [13] as they are prescribed in fixed doses and routine laboratory coagulation monitoring is not required [14–17]. The drugs that have completed phase 3 trials for VTE include: rivaroxaban and apixaban (competitive inhibitors of activated factor X) and dabigatran, (direct inhibitor of thrombin). To date, none of the drugs has a specific antidote [8] potentially rendering their bleeding less amenable to control.

### Recurrence risk and fatality

The risk of recurrent thromboembolic disorders in the 10-year period following an episode of unprovoked VTE ranges between 30 and 50 %. The rate is higher in patients with primary DVT than in those with primary PE. The clinical presentation with a primary PE increases by more than three times the risk of a new PE episode over that with isolated DVT. Baseline parameters that increase this risk are the proximal location of DVT, obesity, male sex and old age, whereas the role of thrombophilia is controversial [18].

Contemporary data from population studies on the incidence and complications of VTE are limited. An observational cohort study was undertaken by Martinez et al., 2014 [19], to estimate the incidence of first and recurrent VTE. The cohort was identified from all patients in the UK Clinical Practice Research Datalink (CPRD). Between 2001 and 2011, patients with first VTE were identified and the subset without active cancer-related VTE observed for up to 10 years for recurrence. 35,373 first VTE events were reported with 16,708 (47.2 %) being unprovoked among 26.9 million person-years of observation. The overall incidence rate (IR) of VTE was matching the previously reported rates at 131.5 per 100,000 person-years and 107.0 (95 % CI, 105.8-108.2) after excluding cancer-associated VTE. DVT was more common in the young whereas PE was more common in the elderly. VTE recurrence occurred in 3671 (Corrected IR 25.2 %). The IR for recurrence peaked in the first six months at around 11 per 100 person-years. It leveled out after three years and then remained at around 2 per 100 person-years. The IRs for recurrences was particularly high in young men [19].

Sex therefore seems to affect the risk of recurrence as it was observed that men have about a 13 % higher risk of recurrence than women [20]. The risk in women with unprovoked first VTE was reported by other investigators to be around 3.2 % [21]. A slightly higher rate was reported by a Japanese registry at 3.9 per 100 patient-years also associated with a higher fatality rate [22].

A cochrane review looked at the recurrence rate in eleven studies with a total of 3716 participants. A consistent and strong reduction in the risk of recurrent venous thromboembolic events was observed during prolonged treatment with VKA (risk ratio (RR) 0.20, 95 % confidence interval (CI) 0.11 to 0.38) independent of the period elapsed since the thrombotic event. In addition, a substantial increase in bleeding complications was observed for patients receiving prolonged treatment during the entire period after randomization (RR 2.60, 95 % CI 1.51 to 4.49) while no reduction in mortality was noted during the entire study period (RR 0.89, 95 % CI 0.66 to 1.21, P = 0.46). It was therefore concluded that treatment with VKA strongly reduces the risk of recurrent VTE for as long as they are used. However, the absolute risk of recurrent VTE declines over time while the risk for major bleeding remains unchanged. Thus, the efficacy of VKA administration decreases over time since the index event [23].

A search for predictors of recurrence to identify candidates for prolonged anticoagulation was conducted and concluded that patients with isolated distal DVT have a significantly lower risk of overall VTE recurrence than did patients with isolated proximal DVT but a similar risk of serious recurrent VTE. Age > 50 years, unprovoked distal DVT, and number of thrombosed veins (more than one) influenced the risk of recurrence and may help to define patients at significant risk of recurrence [24].

Although the incidence of VTE increases logarithmically with age, patients > or = 65 years are less likely to have "unprovoked" VTE than younger patients. Rates of recurrent VTE do not differ significantly between patients 65 years of age or older compared to younger patients [25].

No differences between NOACs and VKA were found regarding recurrent VTE [26].

Recurrence is also often associated with a high fatality rate in certain geographic areas. The case-fatality rate (CFR) for recurrent VTE was reported to be 12.8 % (95 % CI 7.99-19.1) in the Mediterranean region and around 8.41 % (5.15-12.9) in other areas [27].

A relatively high rate and significant mortality associated with recurrence is therefore observed in a proportion of the population that needs to be identified with optimization of the duration of anticoagulation to cover the maximal “at risk” period.

### Bleeding risk

Observational studies have reported a wide range of bleeding risks that differ 40-fold. This variation may be caused by time trends, variation in bleeding definition and study subject selection. The incidence of first-time severe bleeding was reported to be 2.3 per 100 patient-years (95 % confidence interval 1.4 to 3.1) in a Swedish population. Male sex and use of drugs potentially interacting with warfarin were the only independent risk factors of severe bleeding, with hazard ratios of 2.8 and 2.3, respectively [28].

Most of the studies of risk of bleeding on VKAs are derived from randomized controlled trials of highly selected patients followed for less than 1 year. Khan and Datta 2014 [29], reviewed patients in an anticoagulation clinic who were treated for more than one year with a VKA to prevent thrombosis recurrence. It was found that many of these patients had serious comorbidities. The latter is in contrast to many studies in which patients are highly selected. They found the overall rate of bleeding was 10 episodes per 100 person-years. Major bleeding episodes were 5.2 episodes per 100 person-years [29].

In a study conducted to report on bleeding with VKA in a general practice context in the UK, the overall incidence of first-time, idiopathic bleeding was 15.2 per 100 patient-years of current warfarin exposure: the incidence of fatal/hospitalized and referred bleeding was 3.5 and 2.6 per 100 patient-years, respectively [30].

Drug-drug interaction is among the most important determinants of bleeding in patients on warfarin. In a recent study, 16.5 % of patients have developed bleeding complications as a result of drug-drug interactions, correlating with a marked prolongation of their INR [31].

The long experience with warfarin and its reversal in the emergency rooms using oral and intravenous vitamin K, FFP or prothrombin complex concentrate (PCC) in the context of bleeding or urgent surgical interventions underscore to a certain extent its bleeding risks.

The bleeding risk profile of the NOACs seemed initially to be more favorable while maintaining a non-inferiority efficacy versus warfarin. For the populations in the Mini-Sentinel data assessment, the combined incidence rate (Intracranial Hemorrhage [ICH] and Gastrointestinal hemorrhage [GIH] events per 100,000 days at risk) was 1.8 to 2.6 times higher for new users of warfarin than for new users of dabigatran. The incidence rate of GIH events only per 100,000 days at risk was 1.6 to 2.2 times higher for warfarin new users than for dabigatran new users, and the incidence rate of ICH events only per 100,000 days at risk was 2.1 to 3.0 times higher with warfarin than with dabigatran. The results indicate that the observed bleeding rates associated with new use of dabigatran do not appear to be higher than the bleeding rates associated with new use of warfarin [32].

As for the bleeding with rivaroxaban (HR, 0.55; 95 % CI, 0.35-0.89) and apixaban (HR, 0.31; 95 % CI, 0.15-0.62) the anti-Xa inhibitors, a lower incidence of bleeding was observed in comparison to that seen with the use of LMWH-VKA combination followed by VKA. An even lower proportion of patients experienced a major bleeding event during 3 months of anticoagulation with rivaroxaban, apixaban, and low molecular weight herparin-VKA combination (0.49 %, 0.28 % and 0.89 % respectively; 95 % CI) [33].

In other studies comparing NOACs with warfarin bleeding risks was significantly reduced by NOACs: major bleeding by rivaroxaban (RR 0.55; 0.38 - 0.81) and apixaban (RR 0.31; 0.17 - 0.55); major and clinically relevant non-major bleeding by dabigatran (RR 0.63; 0.51 - 0.77), apixaban (RR 0.44; 0.36 - 0.55) and edoxaban (RR 0.81; 0.71 - 0.93). The absolute risk reduction for major bleeding was 1 % for rivaroxaban and apixaban; and for the composite bleeding endpoint 3.2 % for dabigatran, 5.4 % for apixaban, and 1.9 % for edoxaban [26].

Again, bleeding data for the NOACs are derived from the carefully selected patients enrolled in clinical trials. Dabigatran and to a lesser extent, rivaroxaban depend largely on renal excretion for their elimination whereas apixaban has a dual route of excretion. In elderly patients maintained on prolonged NOACs therapy with declining kidney functions and creatinine clearance, it is likely that the bleeding risk would increase logarithmically. Studies specifically conducted on geriatric older, frail population with multiple co-morbidities are therefore needed to evaluate tolerance of NOACs in real life conditions.

In a study on real-life scenario for 1776 rivaroxaban-treated patients, 762 patients (42.9 %) reported 1082 bleeding events during/within 3 days after last intake of rivaroxaban (58.9 % minor, 35.0 % of non-major clinically relevant, and 6.1 % major bleeding according to International Society on Thrombosis and Haemostasis definition). In case of major bleeding, surgical or interventional treatment was needed in 37.8 % and prothrombin complex concentrate in 9.1 %. In the time-to-first-event analysis, 100-patient-year rates of major bleeding was 4.1 (95 % confidence interval 2.5-6.4) for venous thromboembolism patients. In the as-treated analysis, case fatality rates of bleeding leading to hospitalizations were 5.1 % and 6.3 % at days 30 and 90 after bleeding, respectively. The data indicate therefore that, in real life, rates of rivaroxaban-related major bleeding may be marginally lower compared to the reported 5.2 % with VKA [29] and that the outcome may be at least not worse than that of major vitamin K antagonist bleeding [34]. Paradoxically, in a meta-analysis by Holster et al. 2013 [35], looking at gastrointestinal bleeding risks in patients with VTE or acute coronary syndrome on NOACs, among the drugs studied compared to VKA, the OR for apixaban was 1.23 (95 % CI, 0.56_2.73), the OR for dabigatran was 1.58 (95 % CI, 1.29_1.93), the OR for edoxaban was 0.31 (95 % CI, 0.01_7.69), and the OR for rivaroxaban was 1.48 (95 % CI, 1.21_1.82). The overall OR for clinically relevant bleeding in patients taking NOACs was 1.16 (95 % CI, 1.00_1.34) and concluded that Studies on treatment of VTE or acute coronary syndrome have shown that patients treated with NOACs have an increased risk of GIB, compared with those who receive standard care [35].

NOACs are therefore as efficient in the treatment of VTE as VKA but might confer a potential for reduced risk of bleeding complications. Indirect comparisons indicate differences in the risk of clinically relevant bleeding events. Important issues such as monitoring and reversal of anticoagulation are still unresolved, but introduction of NOACs increased the therapeutic spectrum and thereby the potential for individualized therapy [26].

### Benefit/risk ratio of prolonged anticoagulation

Despite the rigid guidelines, whether to continue oral anticoagulant therapy indefinitely after completing 3 to 6 months of oral anticoagulant therapy for "unprovoked" VTE is still an unanswered question in VTE management in real life scenarios. This long-term decision should be based on balancing the long-term mortality risk from recurrent VTE, largely preventable with oral anticoagulant therapy, against the long-term mortality risk of major bleeding, the major complication of oral anticoagulant therapy. There exist important knowledge gaps in estimating the long-term mortality risk of recurrent VTE in patients with unprovoked VTE who discontinue therapy and the long-term mortality risk from major bleeding in those who continue oral anticoagulant therapy. These knowledge gaps are the source of uncertainty for patients and health care providers addressing this important question [36]. These gaps are even wider as the recurrence rates and bleeding risks with the NOACs are still scarce.

Lecumberri et al., 2013 [37], aimed to provide estimates of the case-fatality rate (CFR) of recurrent VTE and major bleeding during anticoagulation in a 'real life' population, and to assess these outcomes according to the initial presentation of VTE and its etiology. The study included 41,826 patients with confirmed VTE from the RIETE registry who received different durations of anticoagulation (mean 7.8 ± 0.6 months). During 27,110 patient-years, the CFR was 12.1 % (95 % CI, 10.2-14.2) for recurrent VTE, and 19.7 % (95 % CI, 17.4-22.1) for major bleeding. During the first three months of anticoagulant therapy, the CFR of recurrent VTE was 16.1 % (95 % CI, 13.6-18.9), compared to 2.0 % (95 % CI, 0–4.2) beyond this period. The CFR of bleeding was 20.2 % (95 % CI, 17.5-23.1) during the first three months, compared to 18.2 % (95 % CI, 14.0-23.2) beyond this period. The CFR of recurrent VTE was higher in patients initially presenting with PE (18.5 %; 95 % CI, 15.3-22.1) than in those with DVT (6.3 %; 95 % CI, 4.5-8.6), and in patients with provoked VTE (16.3 %; 95 % CI, 13.6-19.4) than in those with unprovoked VTE (5.5 %; 95 % CI, 3.5-8.0). In conclusion, the CFR of recurrent VTE decreased over time during anticoagulation, while the CFR of major bleeding remained stable reducing the benefit/risk ratio [37].

Another report pointed that VTE unrelated to a transient risk factor was associated with increased mortality compared to mortality in patients with a transient risk factor (hazard ratio (HR) 2.81; 95 % CI 1.40-5.62 [38].

Age is an important determining factor for de novo “first unprovoked” VTE. Patients 65 years of age or older have a much higher incidence of VTE compared to younger subjects but are slightly less likely to have an unprovoked event. Their rates of recurrence do not differ significantly when compared to younger patients [25] but they tend to present with more PEs with a higher case fatality [19]. The adjusted rates of major bleeding increase by approximately two-fold, however in older patients. Therefore, advancing age is not a predictor of recurrent VTE but is associated with more PEs yet with a significant increase in major bleeding episodes with VKA use [25].

Based on the reduced benefit/risk ratio noted with time, and although guidelines suggest extended treatment for all patients after unprovoked VTE unless bleeding risk is high, De Jong et al., 2012 [38], emphasize that the long-term risks of recurrent VTE off anticoagulation are uncertain whereas the risk of bleeding associated with anticoagulant therapy increases with age. In the absence of evidence of reduced mortality or improved quality of life with extended anticoagulant treatment, they suggested a limited duration for most patients after a first VTE. Extended treatment being considered based mainly on patient preference [39].

**Fig. 1:**
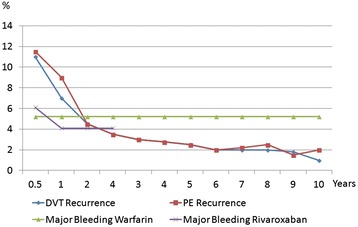
Recurrence of DVT and PE over time after an unprovoked episode compared to the risks of major bleeding with anticoagulation using warfarin or rivaroxaban (modified from Martinez et al., 2014 [19])

Although the use of NOACs seems to tip the balance of the benefit/risk ratio towards more benefit, one promising solution to the current dilemma is risk stratification where unprovoked VTE patients would be categorized as low or high risk for recurrent VTE. Clinical decision making would become less ambiguous and ultimately likely leading to better outcomes [36]. Figure 1 illustrates the recurrence rates of VTE compared to the risks of major bleeding.

### Risk stratification model

VTE associated with active cancer, or a second unprovoked VTE, has a high risk of recurrence and should be treated indefinitely. Furthermore, in cancer-associated VTE recurrences a clinical risk assessment model (RAM) has been presented. By combining 4 clinical patient characteristics (sex, cancer type and stage, history of VTE), the Ottawa score allows stratification of cancer patients according to their VTE recurrence risk. The prediction tool is successfully validated in more than 800 patients from 2 prospective VTE treatment studies [40].

The challenging question of optimal therapy for a first isolated VTE can be addressed by considering the use of risk assessment models [RAMs] or tools.

An increasing role is played by post-baseline parameters such as the ultrasound assessment of residual vein thrombosis and the determination of D-dimer. New scenarios are therefore, being offered through the identification of risk stratification models and by strategies that have the potential to help identify patients in who anticoagulation can be safely discontinued [18].

In an ideal world, genetic testing to determine susceptibility for recurrence could also be used a good stratification tool as multiple genetic SNP analysis is useful in the prediction of recurrent thrombosis [41]. Such techniques are not yet feasible in most centers.

In unprovoked VTE, however, residual vein thrombosis was considered. Residual venous obstruction (RVO) was assumed to improve the stratification of the risk of recurrence after unprovoked deep vein thrombosis (DVT), but results from clinical studies and study-level meta-analyses are conflicting [41].

An early study showed that RVO was associated with a modestly increased risk of recurrent VTE in patients with DVT (unprovoked and provoked). However, RVO did not seem to be a predictor of recurrent VTE in patients with unprovoked DVT following anticoagulation discontinuation [42].

A total of 2527 patients from 10 prospective studies were included. RVO was found in 1380 patients (55.1 %) after a median of six months from a first unprovoked DVT. Recurrent VTE occurred in 399 patients (15.8 %) during a median follow-up of 23.3 months. RVO was independently associated with recurrent VTE (HR = 1.32, 95 % confidence interval [CI]: 1.06-1.65). The association was stronger if RVO was detected early, i.e. at three months after DVT (HR = 2.17; 95 % CI: 1.11-4.25), but non-significant if detected later rendering RVO a weak predictor of recurrence except when performed at 3 months from the initiation of anticoagulants [43]. This observation was also confirmed by an earlier study reporting that RVO at the time of VKA withdrawal was not associated with a statistically significant higher risk of recurrence of VTE; pointing that RVO assessment may not be useful to guide duration of anticoagulation [44, 45]. In the subset of cancer patients, however, (usually not considered as unprovoked VTE), the presence of RVO at attempted discontinuation is associated with an increase in VTE recurrence risk [44, 46, 47].

It was still unknown if residual perfusion defects (PD) on lung scintigraphy are related to recurrent PE but a recent study pointed that there is no significant association found between PD >10 % and VTE recurrence rendering this tool non-predictive of VTE recurrence [48].

The site of DVT was also explored in a complex predictive model, the Vienna model, were proximal/PE were more likely to recur than calf DVT [49] confirming previous reports were also the number of thrombosed veins (more than one) was a marker [24].

Over the past decades, there have been great advances in the understanding of the pathogenesis of VTE through the identification of several inherited and acquired risk factors. However, it is well known that some subjects carrying several risk factors for VTE will never experience a thrombotic episode while other individuals developed recurrent VTEs in their absence questioning the value of thrombophilia screening [50]. It is now almost acceptable that selective screening based on family history of thrombembolism is more cost-effective and clinically valuable than universal screening [51].

As early as 2002, Palareti et al. [52], alluded to the negative predictive value for VTE recurrence of the D-Dimer test performed after 1 month of the discontinuation of oral anticoagulation [52, 53].

The cumulative probability of recurrent VTE at 2 years was 3.7 % (95 % CI, 0.9 %-6.5 %) among patients with D-dimer levels of less than 250 ng/mL compared with 11.5 % (95 % CI, 8.0 %-15.0 %) among patients with higher levels (P = .001) in larger studies [54].

The role of D-Dimer as a risk stratification tool seems however, to be more defined. In a recent study on outpatients with a first unprovoked VTE and after at least 3 months of anticoagulation, serial D-dimer measurements were performed with predefined age/sex-specific cutoffs and were followed for up to 2 years. Of 1010 patients, anticoagulation was stopped in 528 (52.3 %) with persistently negative D-dimer who subsequently experienced 25 recurrences (3.0 % patients-year; 95 % confidence interval [CI], 2.0-4.4 %). It was therefore evident that serial D-dimer measurement is suitable in clinical practice for the identification of VTE patients in whom anticoagulation can be safely discontinued [55]. In a more recent study by Kearon et al. 2014 [55], the risk of recurrence in patients with a first unprovoked VTE who have a negative D-dimer results is not low enough to justify stopping anticoagulation in men but may be low enough to justify the discontinuation in women [56].

Combining D-Dimer in a dyad model with RVO consolidated the value of D-Dimer done at one month [45] and integrating the D-Dimer following the discontinuation of anticoagulation in more complex predictive models have also been attempted [24, 48].

Interestingly D-Dimer was also used as a biomarker during warfarin therapy as high levels of D-Dimer predicted major bleeding, cardiovascular events and all-cause mortality [57].

More integrative predictive models for discontinuation of anticoagulation have been advocated. The MEN-HERDOO_2_ model is based on data prospectively derived by Rodger et al., 2008 to identify patients with less than a 3 % annual risk of recurrent VTE after their first event of idiopathic proximal deep vein thrombosis or pulmonary embolism. Risk factors for recurrent VTE were male sex (the “men” of “Men and HERDOO_2_”), signs of post-thrombotic syndrome, including Hyperpigmentation of the lower extremities, Edema or Redness of either leg, a D-dimer level > 250 μg/L, Obesity (body mass index > 30 kg/m^2^, and Older age (>65 years). There was no combination of clinical predictors that satisfied our criteria for identifying a low-risk subgroup of men whereas women with 0 or 1 risk factor may safely discontinue oral anticoagulant therapy after 6 months of therapy following a first unprovoked venous thromboembolism [58].

The DASH score has also been advocated for, in this scoring system, for each individual subject, the score is calculated based on a sum of scores of four predictors: age, sex, hormone use at the time of the VTE, and D-dimer measured three weeks after stopping oral anticoagulation. A score of +2 is assigned for abnormal D-dimer, +1 for age 50 years old, +1 for male sex, and −2 for hormone use at onset of VTE (among women), generating a D_2_A_1_S_1_H_−2_ score. Therefore, for individuals with an initial unprovoked VTE and a DASH score of 1, the annual VTE recurrence risk is 3.1 percent which justifies stopping anticoagulation after three to six months, assuming (based on approximation of the annual bleeding incidence) that a VTE recurrence rate of less than 5 percent is an acceptable risk. In contrast, a DASH score of 2 is associated with a VTE recurrence risk of 6.4 percent or higher, a risk level sufficiently high to warrant prolonged anticoagulation if the bleeding risk is acceptable [59].

The Vienna score is based, on the other hand on preselected clinical and laboratory variables (age, sex, location of VTE, body mass index, factor V Leiden, prothrombin G20210A mutation, D-dimer, and in vitro thrombin generation) and is difficult to apply in routine clinical settings [60], the use of thrombophilia screen being not universally acceptable as a screening tool for recurrent and even refuted as a stratification tool in another Australian model [61].

Galanaud et al. 2014 [24] used simple predictive variables to conclude that after stopping anticoagulants, patients with isolated distal DVT have a significantly lower risk of overall VTE recurrence than did patients with isolated proximal DVT but a similar risk of serious recurrent VTE. Age > 50 years, unprovoked isolated distal DVT, and number of thrombosed veins influenced the risk of recurrence and may help to define patients at significant risk of recurrence [24].

Although many RAMs have been advocated and seem to help identify patients who would benefit from a more prolonged anticoagulation, none has been widely validated and their use remains in the discretion domain of the treating physicians.

### Can aspirin tip the balance?

The first CRT to address the value of aspirin as an alternative to warfarin in unprovoked VTE following the completion of an initial warfarin therapy was the WARFASA trial which was an investigator-initiated, double-blind study. Patients with first-ever unprovoked venous thromboembolism who had completed 6 to 18 months of oral anticoagulant treatment were randomly assigned to aspirin, 100 mg daily, or placebo for 2 years. The primary efficacy outcome was recurrence of venous thromboembolism, and major bleeding was the primary safety outcome. Venous thromboembolism recurred in 28 of the 205 patients who received aspirin and in 43 of the 197 patients who received placebo (6.6 % vs. 11.2 % per year; hazard ratio, 0.58; 95 % confidence interval [CI], 0.36 to 0.93). One patient in each treatment group had a major bleeding episode. Adverse events were similar in the two groups. It was concluded therefore, that aspirin reduced the risk of recurrence when given to patients with unprovoked venous thromboembolism who had discontinued anticoagulant treatment, with no apparent increase in the risk of major bleeding [62].

In the ASPIRE study published in the same year, aspirin, as compared with placebo, venous thromboembolism recurred in 73 of 411 patients assigned to placebo and in 57 of 411 assigned to aspirin (a rate of 6.5 % per year vs. 4.8 % per year; hazard ratio with aspirin, 0.74; 95 % confidence interval [CI], 0.52 to 1.05; P = 0.09). There was no significant between-group difference in the rates of major or clinically relevant non-major bleeding episodes (rate of 0.6 % per year with placebo vs. 1.1 % per year with aspirin, P = 0.22) or serious adverse events. Aspirin therefore lead to a significant reduction in the rate of major vascular events, with improved net clinical benefit substantiating the earlier evidence of its benefit when it is given to patients after initial anticoagulant therapy for a first episode of unprovoked venous thromboembolism [63].

These two trials, however, were not powered to detect treatment effects for particular outcomes or subgroups. The recently published INSPIRE collaboration used individual patient data analysis of these trials to assess the effect of aspirin versus placebo on recurrent VTE, major vascular events and bleeding. Aspirin reduced recurrent VTE (7.5 % year vs 5.1 % year) including DVT (HR 0.66) and PE (HR 0.66) as well as, as expected, major vascular events. After adjusting for adherence, reduction was 42 % and was more marked in men and older patients. The overall risk of recurrence was reduced by more than one third without any significant increase in the risk of bleeding [64].

The recent data and data analysis of aspirin as alternative agent after the initial anticoagulation in patients with unprovoked first isolated DVT are therefore very encouraging especially if we apply the risk models were male sex and old age are more prone to recurrence but also prone to bleeding. Larger studies are needed to confirm the efficacy and safety of this approach which seems to tip the balance of the benefit/risk of long term pharmacological therapy for these patients.

## Conclusion

The optimal management strategy for long term treatment of idiopathic VTE remains controversial. Cancer-associated VTE and second objectively proven VTE typically mandate a lifelong anticoagulation. It is clear that there must be careful consideration of the benefits of longterm thromboprophylaxis relative to the risk of major hemorrhage for patients with first unprovoked VTE.

CFR of VTE versus major hemorrhage is comparable in the initial acute treatment phase. The CFR of recurrent VTE, however, decreases with time whereas the risk of major hemorrhage remains consistently high for the entire treatment time period.

Risk stratification models may help identifying patients at higher risks of recurrence were anticoagulation seems mandatory from low risk patients.

The historical standard of warfarin as the sole agent of thromboprophylaxis may be undergoing a paradigm shift. The emergence of the NOACs and reconsideration of the benefit of aspirin give us additional clinical tools to make a thoughtful, safe, and evidence based decisions that will serve our patients most effectively.

## Abbreviations

CFR: Case fatality rate
VTE: Venous thromboembolism
DVT: Deep venous thrombosis
PE: Pulmonary embolism
VKA: Vitamin K antagonist
NOACs: Novel oral anticoagulants
RVO: Residual venous obstruction
PD: Perfusion defect
RAM: Risk assessment model
